# The Interplay Between the DNA Damage Response, RNA Processing and Extracellular Vesicles

**DOI:** 10.3389/fonc.2019.01538

**Published:** 2020-01-17

**Authors:** Xiangbing Meng, Shujie Yang, Vanessa J. A. Camp

**Affiliations:** ^1^Department of Pathology, Carver College of Medicine, University of Iowa, Iowa City, IA, United States; ^2^Holden Comprehensive Cancer Center, Carver College of Medicine, University of Iowa, Iowa City, IA, United States

**Keywords:** THRAP3, BCLAF1, RNA processing, extracellular vesicles, R-loops, RBPs, PD-L1

## Abstract

RNA processing was recently found to affect DNA damage response. The RNA processing factors THRAP3 and BCLAF1 play critical role in keeping DNA genomic stability by regulating the transcription, mRNA splicing and export of DNA repair proteins BRCA2, PALB2, Rad51, FANCD2, and FANCL in response to DNA damage. RNA processing factors THRAP3 and BCLAF1 play critical roles in maintaining DNA genomic stability. These factors regulate transcription, mRNA splicing and nuclear RNA export of DNA repair proteins BRCA2, PALB2, Rad51, FANCD2, and FANCL in response to DNA damage. Splicing factors SRSF10 and Sam68 were found to control the DNA damage agent-induced mRNA splicing of transcripts including BCLAF1, BRCA1, BCL2L1, CASP8, CHK2, and RBBP8 to regulate apoptosis, cell-cycle transition and DNA repair. Splicing factors and RNA binding proteins (RBPs) were also found to play a critical role in DNA/RNA hybrids (R-loops) formed during transcription and RNA processing to prevent RNA-induced genome instability. At the same time, DNA repair proteins FANCI and FANCD2 were found to regulate the nuclear localization of splicing factors SF3B1 in the DNA damage response. In addition, tumor-derived extracellular vesicles (Evs) enhanced by chemotherapeutic agents in cancer were found to promote cancer metastasis and drug resistance. Inhibiting Evs from cancer cells significantly reduced cancer metastasis and drug resistance. Furthermore, cross-talk between the DNA damage response and the immune response was observed including the enhancement of the efficacy of immune checkpoint blockade by PARP inhibitors and the effect of PD-L1 on mRNA stability of various mRNAs involved in DNA damage response by acting as a novel RNA binding protein to increase drug resistance in cancer cells. This review will introduce recent progress on the interplay of the DNA damage response, the RNA processing and the extracellular vesicles mediated metastasis.

## Introduction

Generally, RNA processing is not included in DNA damage response network, which is mainly consisted of DNA repair proteins, cell cycle checkpoint regulators, PI3K-like kinases ATM, ATR, or DNA-PK and downstream kinases Chk1 and Chk2. However, recent studies indicate RNA processing directly involves in traditional DNA damage repair mediated by BRCA1 (1) and BRCA2 (2, 3). Many observations indicate that there are connections between DNA damage and immune system activation. Intracellular immune checkpoint protein PD-L1 was fond to regulate DNA damage response by acting as RNA binding proteins to regulate many DNA repair proteins (4). DNA damage also can activate immune system (5–7). In this review, we will introduce recent progression on how RNA processing cross-talk with cellular response to DNA damage and the connections between immune system with cellular response to DNA damage including how immune checkpoint protein PD-L1 regulates cellular response to DNA damage and how DNA damage can active immune system.

### RNA Processing Factors Also Function in Maintaining DNA Genomic Stability

**Figure 1:**
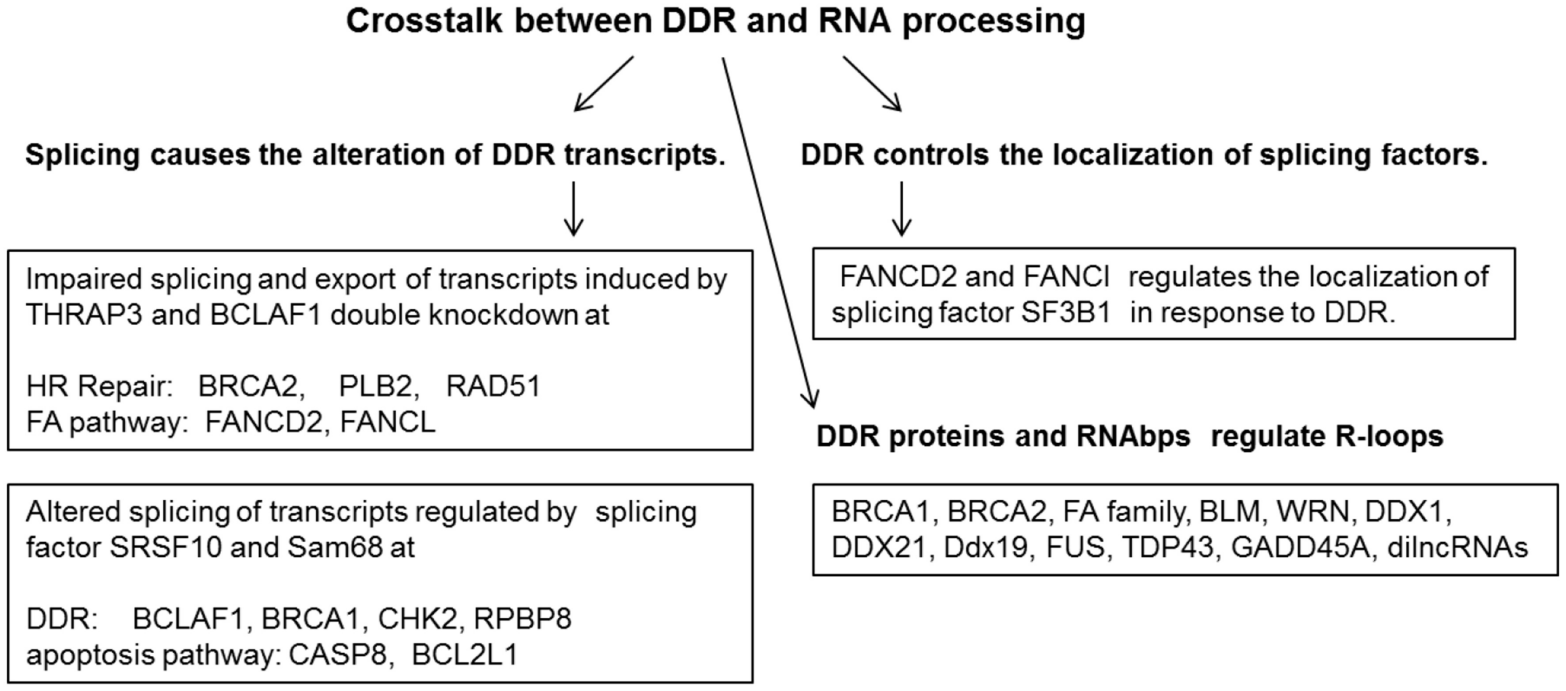
DNA damage response and repair proteins and RNA binding proteins act coordinately to maintain genome stability.

RNA-processing factors function in the maintenance of genome stability; they regulate mRNAs encoding for DNA repair proteins or directly involve in DNA damage responses by interacting with DNA repair proteins. For example, RBM14 is an RNAbp and joins the PARP-dependent DSB repair by interacting with PARP1 (8). Other RNA-binding proteins including FUS/TLS, EWS, TARF15, and some hnRNPs also play important roles in the PARP-dependent DSB repair process (8, 9). mRNA splicing factor hnRNP C is another example required for PALB2/BRCA2 nucleoprotein complex function in DNA repair (3). Knockdown of hnRNP C caused the expression reduction of DNA repair proteins including BRCA1, BRCA2, RAD51, and BRIP1 at both the mRNA level and the protein level (10). BCLAF1 (1) is a BRCA1 binding partner at the BRCA1-mRNA splicing complex induced by DNA damage, which was named as a Bcl2-associated transcription factor to promote apoptosis. BRCA1/BCLAF1 target genes include *ATRIP, BACH1*, and *EXO1* (1). Besides BCLAF1, the DNA damage-induced BRCA1 protein complex includes BRCA1, Prp8, U2AF65, U2AF35, and SF3B1 (1). Depletion of BRCA1, BCLAF1, and U2AF65 increases sensitivity to DNA damage and causes defective DNA repair. A high incidence of somatic mutations of BCLAF1, U2AF65, U2AF35, SRSF2, SF3A1, SF3B1, and PRPF40B at the BRCA1/BCLAF1 mRNA splicing complex was reported in various cancer types (1). Most transcription and pre-mRNA splicing processes are inhibited in response to DNA damage. However, transcription, pre-mRNA splicing and mRNA exportation from the nucleus are active in response to DNA damage for DNA damage response (DDR) genes including BRCA2, PALB2, Rad51, FANCD2, and FANCL (11). These genes are required for DNA damage repair to maintain genomic stability and are regulated by RNAbps THRAP3 and BCLAF1 in response to DNA damage. Depletion of both BCLAF1 and THRAP3 leads to the reduction of mRNA splicing, downregulation of the export of BCLAF1/THRAP3 target genes, and the loss of their encoded proteins compared to mild effects by depletion of THRAP3 or BCLAF1 alone (Figure 1) (11).

#### Splicing Factors and RNA Helicases Are Involved in Cellular Responses DNA Damage

During the DNA damage response, splicing factors and RNA helicases play integral roles in gene expression. mRNA interactome capture was utilized to identify proteins that were highly enriched in mRNA metabolic processes and components of the nucleolar proteome, including several RNA helicases DDX5/p68, DDX1, SLFN11, and DDX3X (9). DDX54 is one of the 266 RBPs in the DDR proteins with increased binding to poly (A)+ RNA upon IR exposure (9). The interaction of DDX54 with specific proteins of core spliceosomal complexes B (CDC40), C(DDX41), and U2 snRNP including SF3B1, DDX42, U2AF1, and DHX8 was increased upon IR exposure (9). Another example of RNAbp in cellular responses to DNA damage is MFAP1 (microfibrillar-associated protein 1), a spliceosome-associated factor. MFAP1 depletion induced the increase of γH2AX foci and DNA breaks by causing alterations of mRNA splicing and gene expression of target genes involved in cellular responses to DNA damage (12).

### DNA Damage Induces the Alterations of RNA Splicing of Many Transcripts Involved in Genomic Stability Maintenance

DNA damage induced by oxaliplatin was found to change the binding and activity of several regulatory RNA binding proteins including SRSF10, hnRNP A1/A2, and Sam68 on the Bcl-x pre-mRNA to alter splice site selection and to increase the level of pro-apoptotic Bcl-xS (13, 14). These RNA binding proteins also collaborate to drive the DNA damage-induced splicing alteration of several transcripts involved in cellular response to DNA damage including BCLAF1, BRCA1, BCL2L1, CASP8, and CHK2 (Figure 1) (13, 14). Mutations of the RNA processing factors result in the increase of spicing isoforms of DNA repair proteins including BARD1β, FANCEΔ4, and BRCA1-Δ11q in cancers. BRCA1-associated RING domain protein 1 (BARD1) splice variant (SV), BARD1β, can sensitize colon cancer cells to poly ADP ribose polymerase 1 (PARP-1) inhibition by impairing BRCA1 mediated DNA homologous recombination repair (15). FANCE splice isoform (FANCEΔ4) impaired mono-ubiquitination of FANCD2 and FANCI, which inhibits the FA-BRCA pathway (16). A BRCA1-Δ11q splice variant lacking part of exon 11 still contributes to drug resistance to PARP inhibitors and cisplatin. Spliceosome inhibitors can reduce BRCA1-Δ11q levels and increase sensitivity to PARP inhibitors and cisplatin in cancer cells carrying exon 11 mutations of BRCA1 (17).

### DNA Repair Proteins Function to Prevent Co-transcriptional R-loop-Associated DNA Damage

RNA–DNA hybrids (R-loops) have been associated with genomic instability in human diseases including cancer and neurological diseases. RNases H are a family of endonucleases that hydrolyze RNA residues in RNA/DNA hybrids to prevent the accumulation of R-loops for the maintenance of genome stability (18). The ssDNA-binding protein replication protein A (RPA) interacts with RNaseH1 at R loops in cells. RPA acts as a sensor of R loops and a regulator of RNaseH1 in suppression of genomic instability (19). Genome-wide RNA-loops are studied by S9.6 antibody CHIP against RNA–DNA hybrids and RNAse H1 R-ChIP. A catalytically inactive RNASEH1 that can bind RNA–DNA hybrids but not resolve them is used in RNAse H1 R-ChIP (18). In contrast to the S9.6 antibody, RNASEH1 has a higher affinity for RNA-DNA hydrids (20). Using S9.6 antibody coupled to mass spectrometry, SRSF1, FACT, and Top1, were identified as R-loop-associated factors. DHX9 helicase promotes R-loop suppression and transcriptional termination. Endonuclease RNase H and helicases DHX9 (20) and SETX are known to resolve the R-loop (21). The RNA/DNA hybrid interactome is a useful resource to study R-loop biology (22). R-loops at CTG.CAG tracts are vulnerable to cause DNA instability (22–25). Enhanced R-loops formation are observed at gene-specific repeat expansions in many genetic disorders such as Huntington's disease [CAG repeats], and fragile X mental retardation or fragile X syndrome (FXS). These well-known neurological diseases are associated with abnormal R-loops accumulation at trinucleotide repeat (22–25). Splicing factors and RNA binding proteins (RBPs) play critical role in DNA/RNA hybrids (R-loops) to prevent RNA-induced genome instability (26). Although no clear mechanisms have been identified, many DNA repair proteins, RNA binding proteins and long non-coding RNAs are involved in suppression R-loops formation as shown in Table 1.

**Table 1:** Known factors involved in R-loops.

**Factors**	**Function**
BRCA1 and SETX complex	Suppresses R-loop associated DNA damage
BRCA2 and PAF1	Prevent R-loops accumulation
FA pathway	Prevent R-loops accumulation
RECQ like helicases Sgs1and BLM	Regulate R-loop-associated genome instability
WRN	Prevents R-loop-associated genomic instability
RNA helicases DDX1, DDX21, and Ddx19	Reduce R·loops formation
RNA processing proteins FUS and TDP43	Inhibit R loops-associated DNA damage
GA0045A	R-loops dependent TET1 binding CpG islands at promoters
Long non-coding RNAs (dilncRNAs)	Required for R-loop-driven DNA damage repair

#### The BRCA1 and SETX Complex Suppresses R-loop-Associated DNA Damage

Senataxin (SETX) is a RNA/DNA helicase and a BRCA1 interacting protein identified by yeast two hybrid assays and MS- based BRCA1/protein interaction screens (21). Knockout SETX gene leads to a defect in reproduction in male mice. Mutations of SETX is found in two distinct neurological disorders including ataxia with oculomotor apraxia type 2 (26) and a juvenile form of ALS (27). BRCA1 and SETX complex is recruited to suppress co-transcriptional R-loop-associated DNA damage (21). A deficiency in BRCA1/SETX complex results in unrepaired ssDNA breaks and increases of γ-H2Ax signal.

#### Inactivated BRCA2 and Depleted PAF1 Cause the R-loops Accumulation

R-loops are frequently found in BRCA2-deficient cancer cells. BRCA2 is involved in the release of RNA polymerase II (RNAPII) from promoter-proximal pausing (PPP) sites. BRCA2 inactivation decreases RNAPII-associated factor 1 (PAF1) recruitment and impedes nascent RNA synthesis. PAF1 depletion also causes the R-loop accumulation (2, 3).

#### The FA Pathway Plays a Role in Preventing R-loop Accumulation

The FA pathway prevents R loop accumulation that hinders replication fork (RF) progression and results in DNA breaks.

FANCD2 foci increase in untreated and MMC-treated cells defective in FANCD2 or FANCA indicates that the FA functions at R loop. FANCD2 was found to interact and recruit RNA processing (28–30) enzymes hnRNPU and DDX47 to R-loops during mild replication stress (33). BRCA2/FANCD1 and FANCD2/FANCI were found to protect stalled replication forks, indicating that the Fanconi Anemia (FA) pathway may take a role in preventing R loop-dependent genome instability. The Fanconi anemia (FA) pathway is critical to repair inter-strand DNA cross-links (ICLs). However, a 5′ exonuclease, SAN1, is involved in ICLs independent of the FA pathway. Knockout of SAN1 increases sensitivity to ICLs. SAN1 was found to interact with senataxin (SETX) to resolve R-loops to prevent cross-link sensitivity (28–30).

#### R-loop-Associated Genome Instability Is Regulated by RECQ-Like Helicases Sgs1 and BLM

Sgs1 is the ortholog of human Bloom's syndrome helicase BLM in yeast. The loss of *SGS1* increases R-loop accumulation. BLM has been confirmed in suppressing R-loop in Bloom's syndrome fibroblasts or by depletion of BLM in human cancer cells (31).

#### WRN Is a Regulator for R-loop-Associated Genomic Instability

Werner syndrome (WS) is a rare, autosomal recessive disorder characterized by the appearance of premature aging caused by deficiency of Werner protein (WRN). WRN deficiency sensitizes cells to replication- transcription collisions and promotes accumulation of R-loops. WS cells show impaired ATR- mediated CHK1 activation to mild replication stress. WS cells prevent chromosomal instability by ATM mediated activation of CHK1 (32).

#### RNA Helicases DDX1, DDX21, and Ddx19 Are Involved in Reducing R-loops

RNA helicase DDX1 is necessary to maintain the single-stranded DNA generated by end resection. DDX1 plays a role in resolving RNA-DNA structures accumulated at sites of active transcription with DSBs (33). Knockdown of SIRT7 as well as depletion of DDX21 leads to the increased formation of R loops and DNA double-strand breaks, indicating that DDX21 and SIRT7 mediated deacetylation of DDX21 cooperate to prevent R-loop accumulation (34). The nucleopore- associated mRNA export factor Ddx19 was activated by ATR/Chk1 and re-localized to the nucleus to remove nuclear R-loops upon replication stress or DNA damage. Ddx19 resolves R-loops *in vitro* via its helicase activity (35).

#### RNA Processing Proteins FUS and TDP43 Are Involved in R-loop-Associated DNA Damage

FUS and TDP43 are linked to Amyotrophic lateral sclerosis (ALS), a progressive motor neuron dysfunction disease. FUS or TDP43 depletion leads to an accumulation of transcription- associated DNA damage and increased sensitivity to a transcription-arresting agent. FUS or TDP43 normally contribute to the prevention of transcription-associated DNA damage (36).

#### GADD45A Is Involved in R-loops Dependent TET1 Binding CpG Islands at Promoters

R-loops are enriched at CpG islands (CGIs) to regulate chromatin states. GADD45A (growth arrest and DNA damage protein 45A) is an epigenetic R-loop reader to recruit the demethylation machinery at promoter CGIs. GADD45A binds to R-loops and recruits TET1 (ten-eleven translocation 1) to promote DNA demethylation at the promoter of tumor suppressor TCF21. The antisense long non-coding (lncRNA) TARID (TCF21 antisense RNA inducing promoter demethylation) forms an R-loop at the TCF21 promoter and the binding of GADD45A to the R- loop triggers local DNA demethylation and TCF21 expression. Thousands of R-loop-dependent TET1 binding sites at CGIs is identified in embryonic stem cells by genomic profiling (37).

#### Long Non-coding RNAs (dilncRNAs) Are Required for R-loop-Driven DNA Damage Repair

Damage-induced long non-coding RNAs (dilncRNAs) are transcribed from broken DNA ends to pair with the resected DNA ends, form DNA:RNA hybrids and promote homologous recombination (HR) repair by contributing to the recruitment of the HR proteins BRCA1, BRCA2, RNase H2, and RAD51. BRCA2 mediates the localization of RNase H2 to DSBs by directly interacting with RNase H2 (38).

### DNA Repair Proteins Control the Nuclear Distribution of Splicing Factors in Replication Stress

Both FANCD2 and FANCI were co-purified with SF3B1 and yielded strong signals of interaction with SF3B1 in the nucleus in proximity ligation assay (PLA) (39). FANCI and SF3B1 yielded strong PLA signals throughout the cell cycle, whereas PLA signals between FANCD2 and SF3B1 were restricted to the chromatin of interphase cells (39). Therefore, it is hypothesized that FANCI associates with and regulates the dynamics of the nucleoplasmic pool of SF3B1, whereas FANCD2 associates with the chromatin-bound pool of SFs.

## Tumor-Derived Extracellular Vesicles Affect Bystander Cells in Tumor Micro-Environment

Tumor-derived Evs secreted from cancer cells treated with chemotherapy carry distinct type of damage-associated molecular patterns (DAMPs) that activate innate immune cells including natural killer (NK) cells. Stress-induced ligands from tumor-derived Evs bind with activating receptor NKG2D to activate NK cells in the tumor microenvironment (40). Activated NK cells promote the clearance of drug-treated tumor cells (40). The Evs is necessary for the RNA clearance step in homologous recombination repair of DNA double-strand breaks (DSBs). Chemotherapy stress promotes extracellular vesicles (Evs) secretion from tumor cells. The released Evs from cells treated with cisplatin were found to induce invasion and increased resistance to cisplatin via p38 and JNK signaling when taken up by bystander cells in tumor microenvironment. Evs uptake inhibitors heparin, amiloride, and dynasore were shown to prevent Evs-mediated adaptive response and sensitize cells to cisplatin (41). MiR-21 in the exosomes released from cisplatin- resistant oral cavity squamous cell carcinoma (OSCC) cells was reported to decrease the DNA damage signaling in response to cisplatin and increase drug resistance to cisplatin by targeting PTEN and PDCD4 (42). Annexin A6 enriched tumor-derived Evs secreted from cancer cells treated with chemotherapeutic compounds taxanes and anthracyclines were found to promote cancer metastasis to lung by inducing the activation of NFκB and CCL2. Inhibiting annexin A6 in Evs from cancer cells significantly reduced cancer metastasis (43). Exosomes generated from breast cancer cells lead to the generation of reactive oxygen species, DNA damage response, and the stabilization of p53 and autophagy in primary mammary epithelial cells (44). Exosomes released by ovarian cancer regulate intercellular communication between tumor cells and local immune cells, cancer-associated fibroblasts and normal stroma, within the tumor microenvironment to accelerate pre-metastatic niche formation and metastatic invasion (45). Preoperative administrations of the non-steroidal antiinflammatory drug ketorolac and/or resolvins induced T cell responses and eliminated micrometastases in multiple tumor-resection models. Ketorolac and resolvins exhibited synergistic antitumor activity (46). A similar observation was also found in leukemia. Exosomes secreted from acute myeloid leukemia (AML) cells create a leukemic niche at the bone marrow (BM) to promote leukemic cell proliferation by inducing DKK1 and suppress normal hematopoiesis through exosome secretion. Disruption of exosome secretion delayed leukemia development by targeting the exosome release regulator Rab27a in AML cells (47).

## Crosstalk Between the DNA Damage Response and Immune Checkpoint Inhibition

### PD-L1 (B7-H1) Regulates the DNA Damage Response

**Figure 2:**
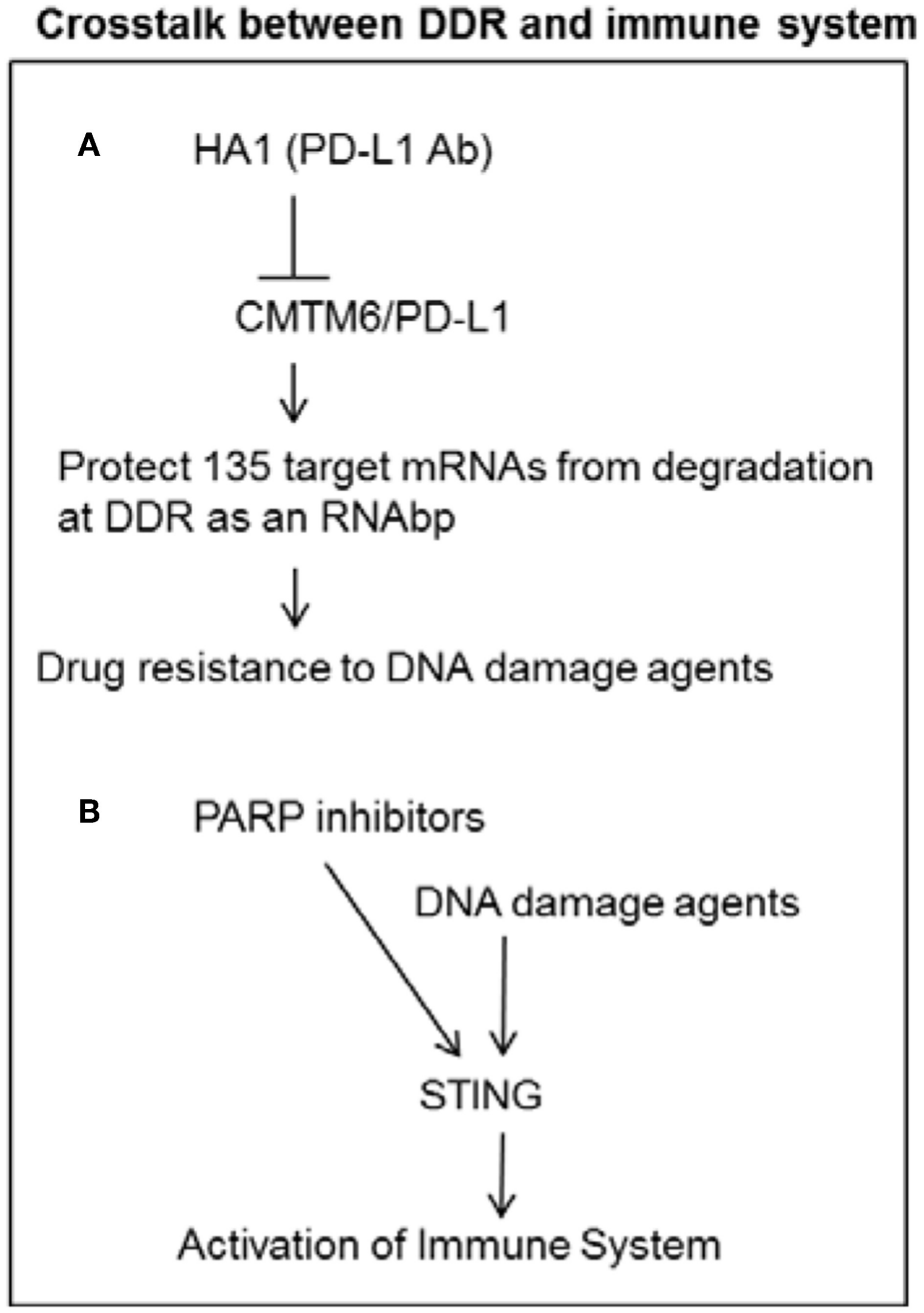
Crosstalk between DDR and immune system. **(A)** PD-L1 can increase mRNA stability of DNA damage response genes as a RNA binding protein in cancer cells. PD-L1 antibody H1A can increase sensitivity to DNA damage agents by reducing PD-L1 mediated stability of DDR transcripts. **(B)** DNA damage agents and PARP inhibitors can induce STING pathway to activate immune system.

PD-L1 has been well-known as immune checkpoint inhibition to the activation of T cells by interacting with PD1. PD-L1 was recently found as a novel RNA binding protein to increase drug resistance in cancer cells by increasing mRNA stability of various mRNAs encoding for proteins involved in DDR and repair (4). Luo lab reported that PD-L1 acts as an RNA binding protein to protect target RNAs from degradation by interacting with EXOSC10 and EXOSC4, which are key components of the RNA exosome (4). Knockdown of PD-L1 by small hairpin RNAs (shRNAs) increases sensitivity to the chemotherapy agent, cisplatin. Knockdown of PD-L1 also increases sensitivity to ionizing radiation (IR) (4). Genome-wide RNA transcripts interacting with PD-L1 were identified by the crosslinked RIP sequencing (RIP-seq) by PD-L1 antibody. PD-L1 knockdown on the alteration of gene expression in genome wide was identified by comparing control and PD-L1 knockdown cells by RNA sequencing (RNA- seq). About 135 genes were found to be enriched in both datasets of the RNA-seq analysis and RIP-seq analysis, including ATM, BRCA1, and FANCL and other genes involved in cellular responses to DNA damage metabolic, transcriptional, and protein modification pathways (4). A PD-L1 antibody, H1A, was developed to destabilize PD-L1 by disrupting the PD-L1 stabilizer CMTM6. This disruption resulted in PD-L1 degradation through the lysosome and increased sensitivity to radiotherapy and cisplatin (4). These studies indicate that targeting intracellular PDL1 may enhance the efficacy of chemotherapy or radiotherapy by overcoming PDL1 mediated drug resistance (Figure 2).

### Activation of Immune System by DNA Damage Response

#### Immune System Is Activated by PARP Inhibitors

Recent studies show that PARP inhibitor or Chk1 inhibitor promotes antitumor immunity of PD-L1 blockade in NSCLC. PARP inhibitor selectively triggers anti-tumor immunity in ERCC1- or BRCA-defective contexts, indicating that PARP inhibitors might promote therapeutic effects by inhibiting DNA damage repair and activating anti-tumor effect in populations with DNA repair defect (Figure 2) (48, 49). PARP inhibitor was also found to trigger the STING-dependent immune response independent of BRCAness (50).

#### Activation of Immune System by DNA Damage Activated STING

In addition to causing the activation of cell cycle checkpoint and DNA repair and the induction of cell death, DNA damage response network induced by chemotherapy and radiotherapy can also activate the immune system. Damaged cancer cells secrete type I interferons and proinflammatory cytokines transcriptionally activated by IRF3 or NFB. The cytosolic damaged DNA from micronuclei can be recognized by the DNA sensor cGAS (cyclic guanosine monophosphate adenosine monophosphate synthase) to activate type I interferons by STING/TBK1/IRF3 pathway (5–7). Homologous recombination repair protein RAD51 also plays a role in initiating immune signaling by preventing the fragmented nascent DNA accumulates in the cytoplasm and initiation of the STING-induced innate immune response (51). Etoposide-induced DNA damage can induce the activation of NF-κB by an alternative STING- dependent and cGAS-independent pathway. The alternative STING signaling pathway includes the DNA damage response proteins ATM (ataxia telangiectasia mutated), PARP1 (poly-ADP-ribose polymerase 1), DNA sensor IFI16 (interferon- inducible protein 16), Tp53, and the E3 ubiquitin LIGASE TRAF6 (52). The efficacy of immune checkpoint blockade (ICB) is enhanced by ATM inhibition and further potentiated by radiation in pancreatic cancer (52).

## Conclusion

In summary, there are cross-talks between cellular responses to DNA damage, RNA processing, and the extracellular vesicles related to immune checkpoint inhibition. RNAbps involved in RNA processing play critical roles in maintaining DNA genomic stability by regulating the transcription, mRNA splicing, and export of DNA repair proteins. On the other hand, DNA repair proteins can regulate the nuclear distribution of splicing factors in response to DNA damage. Splicing factors, RNAbps, and DNA repair proteins also work coordinately to prevent RNA-induced genome instability by resolving R-loops formed during transcription and RNA processing. Cross-talk between the immune response and cellular responses to DNA damage includes the enhancement of the effect of immune checkpoint inhibitors by PARP inhibitors or STING pathway. Tumor-derived Evs enhance cancer metastasis and drug resistance partially due to PD-L1 delivered from tumor-derived Evs, which acts as a novel RNA binding protein to increase drug resistance in cancer cells by affecting mRNA stability of various mRNAs involved in cellular response to DNA damage.

## Author Contributions

XM, SY, and VC wrote the manuscript.

### Conflict of Interest

The authors declare that the research was conducted in the absence of any commercial or financial relationships that could be construed as a potential conflict of interest.

## Abbreviations

FUS/TLS: fused in sarcoma/translocated in sarcoma
EWS: Ewing sarcoma
TARF15: TATA box-binding protein-associated factor 68 kDa
hnRNPs: heterogeneous nuclear ribonucleoproteins
MFAP1: microfibrillar-associated protein 1
IRF3: interferon regulatory factor 3
STING: STimulator of Interferon Genes
TBK1: TANK binding kinase 1
IRF3: interferon regulatory factor 3
Evs: extracellular vesicles
RNAbps: RNA binding proteins
R-loops: DNA/RNA hybrids.
